# Acute myocardial infarction and pulmonary tuberculosis in a young female patient: a case report

**DOI:** 10.1186/1757-1626-1-246

**Published:** 2008-10-17

**Authors:** Aurora Bakalli, Behxhet Osmani, Lulzim Kamberi, Ejup Pllana

**Affiliations:** 1 University Clinical Center of Kosova, Department of Cardiology, Prishtine, Kosove; 2 University Clinical Center of Kosova, Department of Pulmology and Pneumophtysiology, Prishtine, Kosove

## Abstract

**Introduction:**

Tuberculous coronaritis is known to be a very rare phenomenon, although *Mycobacterium tuberculosis*, as some other infectious agents, may also act in the coronary vessels by activating the inflammatory mechanism of atherosclerosis. The association between active pulmonary tuberculosis and acute myocardial infarction has not been reported for around three and a half decades.

**Case presentation:**

We presented here a case of a young, 30 year old, Caucasian woman who presented to Emergency Ward with severe chest pain, ECG and enzyme profile typical for acute myocardial infarction. Chest X-ray displayed high intensity shades in the left lung field, which after additional laboratory tests were shown to be due to active pulmonary tuberculosis.

**Conclusion:**

As the patient did not have any other known coronary artery disease risk factors, we held responsible *Mycobacterium tuberculosis *for occurrence of acute myocardial infarction in this young female patient. We believe that the presentation of this rare case of myocardial infarction in a patient with active pulmonary tuberculosis should encourage researchers to investigate the potential role of *Mycobacterium tuberculosis *in pathogenesis of coronary heart disease.

## Introduction

As in most developing countries, tuberculosis remains a major health problem in Kosova. Tuberculosis is mainly manifested as pulmonary TB, but it affects other organs as well. Tuberculous angitis of small vessels is often associated with tuberculous inflammation and it may reach the blood vessel from inside or outside. Tuberculous arteritis of coronary arteries is a rare phenomenon [1]. However, *Mycobacterium tuberculosis *may be one of the several infectious agents that plays a role in the inflammatory mechanism of the atherosclerotic process [2].

We describe here a case of a young woman presenting with acute onset of chest pain, ST elevation on ECG, elevated cardiac enzymes and active pulmonary tuberculosis. These manifestations are likely due to the involvement of *Mycobacterium tuberculosis*.

## Case presentation

A 30 year old woman presented to the Emergency Ward after mid-night, complaining of severe, squeezing chest pain, radiating to the left arm. This discomfort started approximately 5 hours prior to admission. Associated symptoms included nausea and fatigue. Patient also noted that for a few months has had productive cough and night sweats. Patient suffered from mild arterial hypertension for six years, since her first pregnancy and was not under antihypertensive medication. She underwent cholecystectomy 4 years ago. Patient did not have a history of diabetes mellitus, hyperlipidemia, tobacco use, alcohol, drug abuse or oral contraceptive usage. She also denied having family history of premature coronary artery disease.

Her vital signs were as following: blood pressure 140/90 mmHg; pulse rate 110 beats/min.; respiration rate 17/min.; body temperature 37.5°C. Heart auscultation revealed regular heart rhythm, clear sounds and diastolic murmur 2/6 on the second right intercostal space. On lung auscultation there was normal breathing. Other systems had no remarks on physical examination.

**Figure 1:**
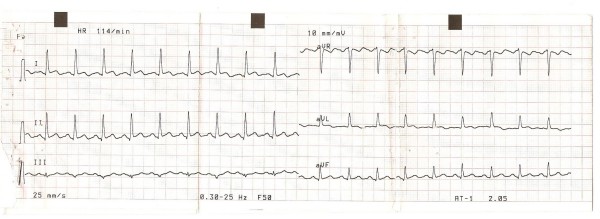
**ECG 1**. Standard leads showing slight ST elevation on D1, D2, D3, aVF and negative T waves on aVL

**Figure 2:**
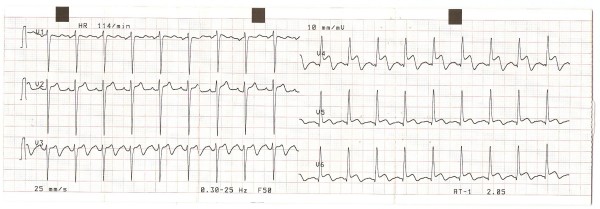
**ECG 2**. Precordial leads showing up to 3 mm ST elevation in leads V3–V6.

ECG showed normal sinus rhythm, heart rate of 110/min., and ST segment elevation of 1–3 mm in inferior and antero-lateral wall leads (figure 1 and 2). Initial enzyme profile that could be obtained in our Emergency Centre Laboratory displayed following results: ALT: 32 IU/l, AST: 72 IU/l, CK: 493 IU/l and CK-MB: 21 IU/l, with upper limit reference values for CK and CK-MB being 110 IU/l and 12 IU/l, respectively. Troponin data could not be attained in our hospital. Subsequent enzyme results showed lower values. CRP level was 20 mg/l, whereas lipid profile revealed the following results: total cholesterol = 4.81 mmol/l; HDL cholesterol = 2.02 mmol/l; LDL cholesterol = 2.41 mmol/l; VLDL = 0.24 mmol/l; and triglycerides = 1.2 mmol/l. All other laboratory findings were within normal reference range.

Based on these findings patient was admitted in the Coronary Unit and was treated as acute myocardial infarction, with analgesics, Oxygen, Aspirin, thrombolytic therapy (Streptokinase), a beta blocker and low dose of ACE inhibitor. Chest X-ray and echocardiography were done that morning.

**Figure 3:**
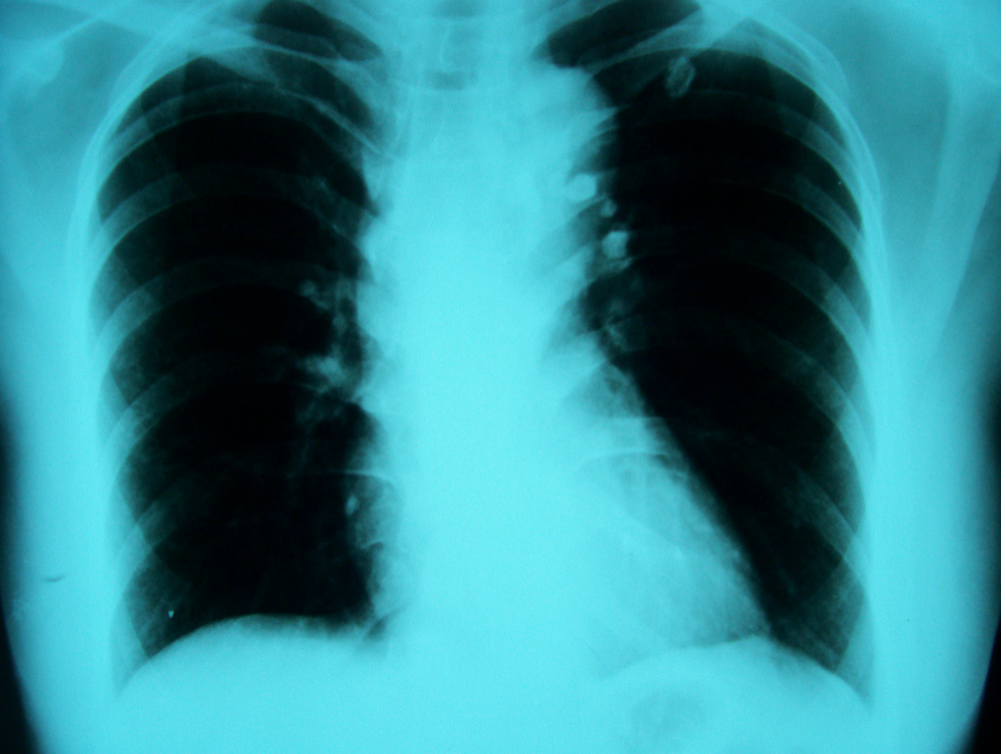
**Chest X-ray**. Chest X-ray revealing solid tissue intensity shades in the left lung field.

Chest X-ray discovered homogenous shades, well defined, of solid tissue intensity, located mainly in the para-tracheal part of the left lung (figure 3).

Echocardiography findings demonstrated: very mild thickening of the septal wall (11,5 mm), thickening of aortic cusps, grade 2 aortic regurgitation, grade 1 mitral regurgitation, diastolic dysfunction of the left ventricle, and hypokinesis of the inferior, anterior and lateral myocardial walls. There was no evidence of echo-free space amid epicardium and pericardium and separation between them was not detected. Pericardium was not thickened. Echocardiography measurements were as follows: Aortic root = 34 mm; Left Atrium = 35 mm; Mid right ventricular diameter = 27 mm; Right atrium diameter = 30 mm; Interventricular septum = 11.5 mm; Posterior wall = 11 mm; Left ventricular end diastolic diameter = 55 mm; Left ventricular end systolic diameter = 38 mm; Fractional shortening (FS) = 31%; Ejection Fraction (EF) = 58%; E/A = 0.8; E wave deceleration time = 240 ms.

**Figure 4:**
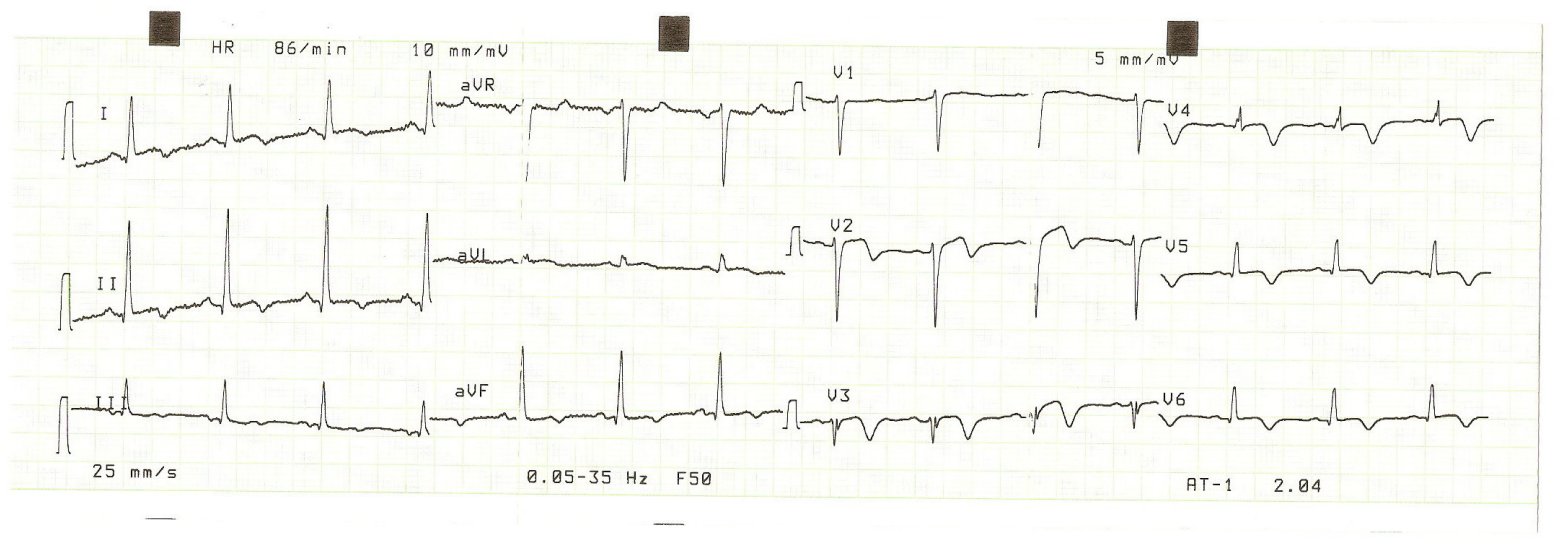
**ECG 3**. ECG of the 10^th ^day, consistent with non-Q-wave myocardial infarction of the inferior and lateral wall, and Q-wave infarction of the anterior wall.

Patient's chest pain went away the next day and did not recur during the 12 day hospitalization period, while weakness, intermittent coughs and night sweats persisted. ECG of the second day showed decrease of ST elevation and terminal T wave inversion in leads V3–V6 and T wave inversion in D2, D3 and aVF. ECG of the third day demonstrated changes consistent with non-Q-wave myocardial infarction of the inferior and lateral wall, and Q-wave infarction of the anterior wall. This ECG pattern remained the same throughout the hospitalization time (figure 4).

Due to chest X-ray abnormalities of unknown nature, a pulmologist was consulted. Hexagon TB test and PPD resulted positive. Patient was diagnosed by pulmologist as Active Pulmonary Tuberculosis and was put under tuberculostatic therapy. Eight weeks later *Mycobacterium tuberculosis *was isolated from the Löwenstein sputum specimen.

Patient was asked to undergo coronary angiography and endocardial biopsy, but due to her socioeconomic status and in the absence of medical insurance system in our country, she was not able to complete it.

At follow-up visit, two weeks after hospital discharge, patient did not experience any chest pain, ECG changes were indistinguishable from the discharge ECG and echocardiography findings did not differ from the ones obtained during hospitalization.

## Discussion

Earlier it was thought that by lowering hypercholesterolemia and hypertension, incidence of coronary heart disease would be reduced significantly. Since this was not the case, researchers had to find new explanations regarding the pathogenesis of atherosclerosis, in particular in coronary arteries.

Recent studies have shown that inflammation is an important factor in coronary atherosclerotic process and the potential role of several infectious agents in atherosclerosis has been recognized. In several studies it is documented the correlation between the *Chlamydia pneumoniae *infection and coronary heart disease [3,4]. However, studies suggest that *Chlamydia pneumoniae *is not a direct cause of the inflammatory process in the coronary artery plaques, but rather it initiates the plaque activation through inflammatory mediators, which get triggered due to the similarity between *C. pneumoniae *antigens and human molecules [5]. There have also been studies showing the link between *Cytomegalovirus *and atherosclerosis [6,7].

As for the relationship between coronary artery disease and tuberculosis, there have been several studies in the 50s until 70s, mainly coming from Russia and India that reported such a relation [1,8-10]. Lately there are very few data in this direction. Recently another study, also coming from Russian authors, holds responsible coronaritis caused by tuberculosis as a cause of myocardial infarction [11]. Ditiatkov et al. reported the prevalence of coronary heart disease, in relation to age and gender, among patients with pulmonary tuberculosis. Coronary artery disease was found in 41 of 491 patients that they studied and it was more frequent among women and older age groups [12]. Kinare et al., on the other hand, reported a case of a 19 year old male who ended fatally due to a large ventricular aneurysm obtained from myocardial infarction caused by tuberculous coronaritis of the left anterior descending branch [1].

A group of researchers attempted to detect Mycobacterium tuberculosis DNA in atherosclerotic plaques [13], encouraged by studies that suggest that vaccination of mice with recombinant Hsp 65 and Hsp 65- rich *M. tuberculosis *resulted in formation of atheromatous plaques [14]. Nevertheless, they failed to isolate Mycobacterium tuberculosis DNA complex in atherosclerotic plaques [13].

Tuberculosis is still a major health problem in Kosova. According to Kosova's National TB Control Program, the incidence of tuberculosis has been growing in post-war Kosova. The number of new TB cases has risen from 34/100,000 in 1989 to 83/100,000 in 2000, thus making Kosova one of the regions with highest tuberculosis incidence in Europe.

Although there are numerous cases of patients with acute myocardial infarction that are free of classic risk factors, we believe that the case we presented draws attention due to our patient's young age and female gender. Myocardial infarction is an uncommon condition in younger women in the absence of coronary risk factors [15].

A major limitation of this case presentation is that coronary angiography could not be performed to confirm the diagnosis of myocardial infarction, or to rule out the possibility of coronary artery anomalies. New cardiovascular imaging techniques, such as Multi-slice spiral computed tomography (MSCT) coronary angiography and coronary magnetic resonance angiography (MRA) could not be carried out either.

## Conclusion

*Mycobacterium tuberculosis *may be involved in the occurrence of myocardial infarction in this young female patient, without any known coronary artery disease risk factors. Our message is that tuberculosis as a possible cause of coronary heart disease should not be forgotten, given that in some parts of the world tuberculosis is not part of history yet.

## Consent

Written informed consent was obtained from the patient for publication of this case report and accompanying images. A copy of the written consent is available for review by the Editor-in-Chief of this journal.

## Competing interests

The authors declare that they have no competing interests.

## Authors' contributions

AB examined, analyzed and interpreted patient data regarding the acute myocardial infarction and was a major contributor in writing the manuscript. BO examined and interpreted patient data regarding pulmonary tuberculosis and offered the data regarding the rate of tuberculosis in Kosova. LK and EP also analyzed the patient data and collected the relevant papers regarding this case. All authors read and approved the final manuscript.
